# Identification of six candidate genes for endometrial carcinoma by bioinformatics analysis

**DOI:** 10.1186/s12957-020-01920-w

**Published:** 2020-07-08

**Authors:** Yiming Zhu, Liang Shi, Ping Chen, Yingli Zhang, Tao Zhu

**Affiliations:** 1 Department of Gynaecology, The First Affiliated Hospital of Zhejiang Chinese Medical University; Zhejiang Provincial Hospital of Traditional Chinese Medicine, Youdian Road, Hangzhou, 310006 Zhejiang China; 2 Institute of Cancer and Basic Medicine (ICBM), Chinese Academy of Sciences, Cancer Hospital of University of Chinese Academy of Sciences, Zhejiang Cancer Hospital, Hangzhou, 310022 Zhejiang China; 3 Department of obstetrics and gynecology, Zhuji People’s Hospital, Zhuji, 311800 Zhejiang China

**Keywords:** Endometrial carcinoma, Bioinformatics analysis, Differentially expressed gene, Pathway, Biomarker

## Abstract

**Background:**

Endometrial carcinoma (EC) is the most common gynecological malignant tumors which poses a serious threat to women health. This study aimed to screen the candidate genes differentially expressed in EC by bioinformatics analysis.

**Methods:**

GEO database and GEO2R online tool were applied to screen the differentially expressed genes (DEGs) of EC from the microarray datasets. Protein-protein interaction (PPI) network for the DEGs was constructed to further explore the relationships among these genes and identify hub DEGs. Gene ontology and KEGG enrichment analyses were performed to investigate the biological role of DEGs. Besides, correlation analysis, genetic alteration, expression profile, and survival analysis of these hub DEGs were also investigated to further explore the roles of these hub gene in mechanism of EC tumorigenesis. qRT-PCR analysis was also performed to verify the expression of identified hub DEGs.

**Results:**

A total of 40 DEGs were screened out as the DEGs with 3 upregulated and 37 downregulated in EC. The gene ontology analysis showed that these genes were significantly enriched in cell adhesion, response to estradiol, and growth factor activity, etc. The KEGG pathway analysis showed that DEGs were enriched in focal adhesion, leukocyte transendothelial migration, PI3K-Akt signaling pathway, and ECM-receptor interaction pathway. More importantly, COL1A1, IGF1, COL5A1, CXCL12, PTEN, and SPP1 were identified as the hub genes of EC. The genetic alteration analysis showed that hub genes were mainly altered in mutation and deep deletion. Expression validation by bioinformatic analysis and qRT-PCR also proved the expression of these six hub genes were differentially expressed in EC. Additionally, significantly better overall survival and disease-free survival were observed with six hub genes altered, and survival outcome in high expression of COL1A1, IGF1, and PTEN patients was also significantly better than low expression patients.

**Conclusions:**

COL1A1, IGF1, COL5A1, CXCL12, PTEN, and SPP1 involved in the pathogenesis of EC and might be candidate genes for diagnosis of EC.

## Introduction

Endometrial carcinoma (EC) is an epithelial malignant tumor of the endometrium and regarded as the most common cancers in female reproductive system [1]. There are about 319,600 new cases diagnosed around the world annually, ranking first in gynecological malignant tumors in high-income countries [2]. In recent years, due to the changes of people's living habits, abusing of informal hormone and sex hormone replacement therapy, the incidence of EC increases significantly, and the population tends to be younger, which poses a serious threat to women health [3, 4]. According to the clinicopathological features, EC can be divided into type I and type II, of which type I (hormone dependent) accounts for about 80% of the EC [5]. Surgical treatment is the main means for EC at present, and appropriate adjuvant therapy is also applied according to the pathological and clinical stages of the tumors. However, about 15–20% of the tumors still relapse after surgical treatment, and the curative effect of the systematic therapy is limited [6]. The lack of early diagnosis and timely treatment to prevent the tumorigenesis or progression particularly held responsible for the most deaths of EC. Therefore, exploring the suitable biomarkers and potential targets for the accurate prediction or diagnosis of EC and to seek the possibilities to improve the therapeutic effect and clinical prognosis of EC patients is urgently needed.

The oncogenesis is an extremely complicated pathophysiological process and regulated by various factors and pathways. Recently, high-throughput sequencing technology is widely used in clinical research, with the pronounced advantages relying on its ability to simultaneously determine the expression information of massive genes one time [7]. This is an ideal method to quickly and effectively screen differentially expressed genes (DEGs) related to certain disease. Based on the massive microarray datasets information stored in a large number of public databases such as The Cancer Genome Atlas (TCGA) database and Gene Expression Omnibus (GEO) database, the target data could be mined, integrated, and reanalyzed by bioinformatics method, which provide useful clues for the research of various diseases [8–10]. It has important value in the discovery of disease related biomarkers, disease mechanisms, the prediction of disease prognosis, and the discovery of targeted drugs. In recent years, many microarray profiling studies also have been performed in EC, but most of the results are limited and usually generated from a single cohort study.

In the present study, we applied integrated bioinformatics analysis for the identification of DEGs as potential biomarkers of EC based on the two mRNA expression profiles from the Gene Expression Omnibus (GEO) database. These analysis may provide possible perspectives for the progression and development of EC as well as identification potential candidate genes for EC diagnose or treatment.

## Material and methods

### Microarray data information

The microarray data used in this study were acquired from GEO database (http://www.ncbi.nlm.nih.gov/gds/), which is a public repository containing the high-throughput gene expression data submitted by research institutions around the world. We chose the mRNA expression profiles of GSE115810 and GSE36389 from the GEO database. The array data of GSE115810 was consisted with 24 EC tissues and 3 normal adjacent mucosa tissues based on the platform of GPL96 [HG-U133A] Affymetrix Human Genome U133A Array. The array data of GSE36389 was consisted with 13 EC tissues and 7 normal adjacent mucosa tissues based on the platform of GPL6480 Agilent-014850 Whole Human Genome Microarray 4x44K G4112F (Probe Name version).

### Differential expression analysis of EC

The two mRNA expression microarray datasets obtained from GEO database were screened by an interactive online tool, GEO2R (www.ncbi.nlm.nih.gov/geo/geo2r). The raw data of microarray datasets were pre-processed via background correction and normalization, then |log_2_fold change (FC)| ≥ 1.5 and *P* value < 0.05 were conducted as the cutoff criteria for the DE mRNAs mining. Then, DE mRNAs from GSE115810 and GSE36389 were intersected and the overlapped mRNAs were identified as the DEGs of EC.

### Integration of protein-protein interaction (PPI) network and hub gene identification

To investigate the interaction associations of DEGs, we applied the DEGs to the Search Tool for the Retrieval of Interacting Genes (STRING, http://string-db.org/), an online tool to explore and analyze PPI interactions of the genes. The DEGs of EC were mapped and the interactions with combined score ≥ 0.4 meet the criterion. Afterwards, a PPI network was constructed and visualized by Cytoscape software (version 3.4.0, http://www.cytoscape.org/). Subsequently, the topological properties including node degree of the DEGs in the PPI network were calculated to further analyze the candidate hub genes from the PPI network.

### Gene functional enrichment analysis of DEGs

Based on the identified DEGs, GO analysis and KEGG pathway analysis of the selected DEGs were carried out by DAVID database (http://www.david.abcc.ncifcrf.gov/). This database can integrate biological data and analytical tools to provide systematic comprehensive biologic annotation information for large scale genes or protein lists (hundreds of ID or protein ID lists) to understand the biological meaning behind numerous genes. GO terms and KEGG pathways with *P* < 0.05 as the cutoff criterion, and the DEGs enriched GO terms and KEGG pathway were ranked by enrichment score (− log_10_ (*P* value)).

### Correlation analysis of the DEGs

Based on raw data of the gene expression profiles from the two microarray datasets we download, correlations between the identified DEGs were also analyzed. Pearson’s correlation analysis was used to conduct the correlation analysis; *P* < 0.05 was considered to be statistically significant.

### Exploring cancer genomics data of hub genes

The cBio Portal for Cancer Genomics (http://cbioportal.org) provides visualization, analysis, and download of large-scale cancer genomics data sets; users can select specific samples to form a dataset and chose certain genes to analyze the copy number aberration frequency and mutation frequency of these genes, as well as the co-expression genes and survival curves. In this study, we used the cBio Cancer Portal to investigate the cancer genomics data of the candidate hub genes. The result of the genomics datasets was shown through a concise and compact genomic alterations graphical summary of the inputted genes list in EC. In addition, overall survival and disease/progression-free survival analyses were also performed by grouping EC data alterations using input of the hub genes.

### Hub gene expression and survival analysis

Online tool UALCAN (http://ualcan.path.uab.edu/index.html) was applied to evaluate the expression profiles of the hub DEGs in EC. The EC expression data was based on The Cancer Genome Atlas (TCGA) database, which generates comprehensive, multi-dimensional maps of the key genomic changes over 30 types of cancer. The Human Protein Atlas (https://www.proteinatlas.org/) online tool was also used to obtain the immunohistochemistry-based map of the hub genes protein expression profiles in normal and EC tissues, and the survival analysis in EC was also performed based on 541 EC patients.

### qRT-PCR validation

To further verify the expression of hub DEGs in EC, we performed qRT-PCR to detect the mRNA level of identified hub DEGs in 13 EC clinical samples and 13 normal endometrial clinical samples from Zhejiang Cancer Hospital, China. This experiment was approved by the Ethical Committee of Zhejiang Cancer Hospital, China, and all participated patients are informed consent and agreement for the research use of the clinical endometrial samples. Total RNA was extracted by the TRIzol Reagent (Sangon Biotech, Shanghai, China), and 1 μg RNA was used to synthesize DNA using SuperScript III Reverse Transcriptase (Invitrogen). Real-time PCR was further performed with SYBR Green Master Mix (Takara, Japan) according to the manufacturer’s protocols. The primers were listed as follows: COL1A1, Forward-5′-AGTGGTTTGGATGGTGCCAA -3′; Reverse-5′-GCACCATCATTTCCACGAGC -3′; IGF1, Forward-5′-AGTGCTGCTTTTGTGATTTCTTGA-3′; Reverse-5′-ACCCTGTGGGCTTGTTGAAA -3′; COL5A1, Forward-5′-CTGACAAGAAGTCCGAAGGG -3′; Reverse-5′-CCACATAGGAGAGCAGTTTCC-3′; CXCL12, Forward-5′-GAAAGCCATGTTGCCAGAGC-3′; Reverse-5′-CTCTCACATCTTGAACCTCTTGT-3′; PTEN, Forward-5′- TCCCAGACATGACAGCCATC-3′; Reverse-5′-TGCTTTGAATCCAAAAACCTTACT-3′; SPP1, Forward-5′-AGCAGCTTTACAACAAATACCCAG- 3′; Reverse-5′-TTACTTGGAAGGGTCTGTGGG- 3′; GAPDH, Forward-5′-AATGGGCAGCCGTTAGGAAA-3′; Reverse-5′-GCCCAATACGACCAAATCAGAG-3′. GAPDH were used as an internal control and relative gene expressions were calculated with the 2^−ΔΔCt^ method.

### Statistical analysis

Statistical analysis was performed using SPSS Statistics software (version 22.0). Two-tailed Student’s *t* test was used for calculating the difference between two groups. *P* value < 0.05 was considered statistically significant.

## Results

### DEGs identification

**Fig. 1:**
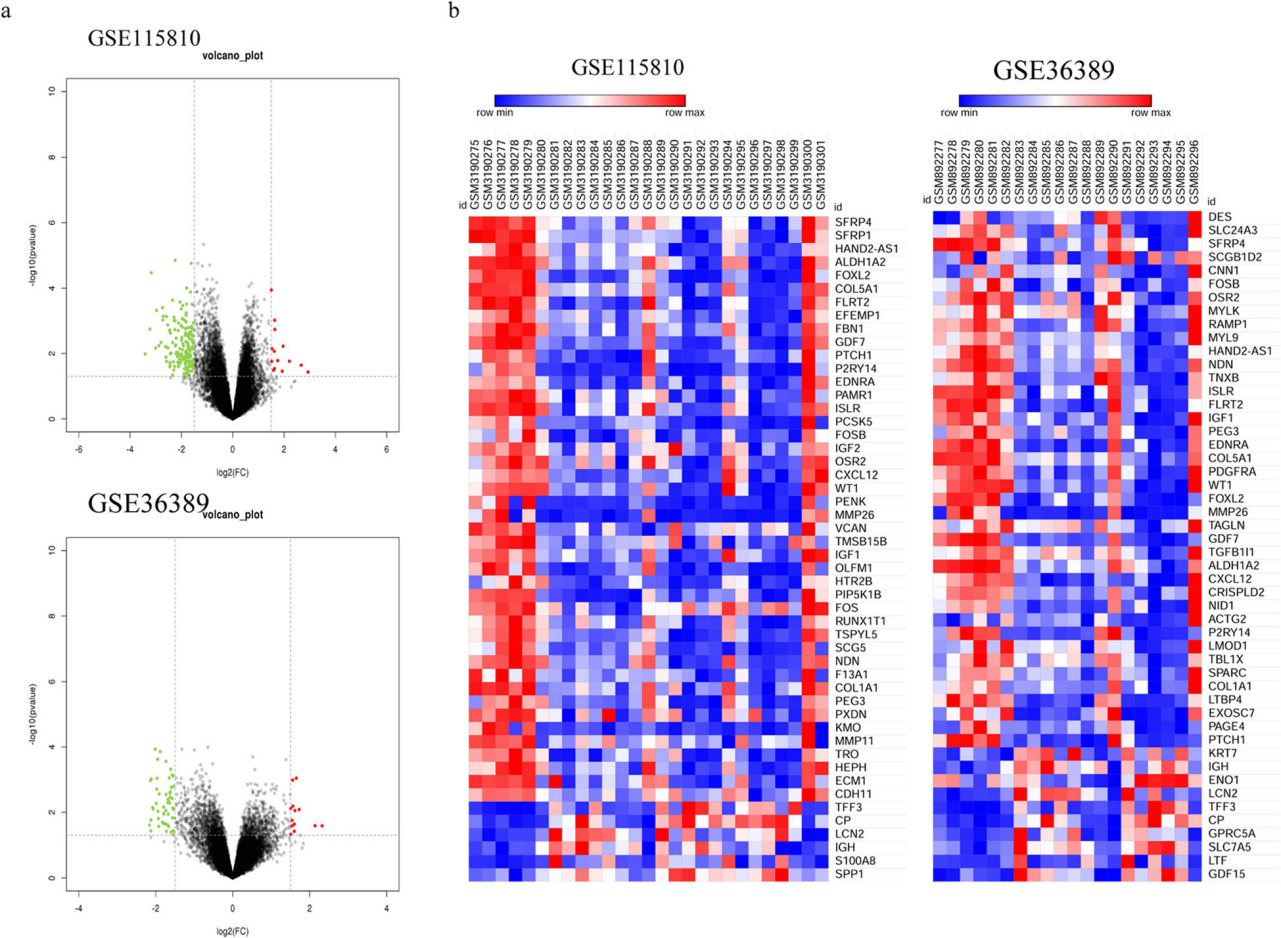
Volcano plot (**a**) and heat map (**b**) of GSE115810 and GSE36389. In the volcano plot, red plot represents upregulated gene, green plot represents downregulated gene, and black plot represents gene without significant expression in microarray dataset

**Fig. 2:**
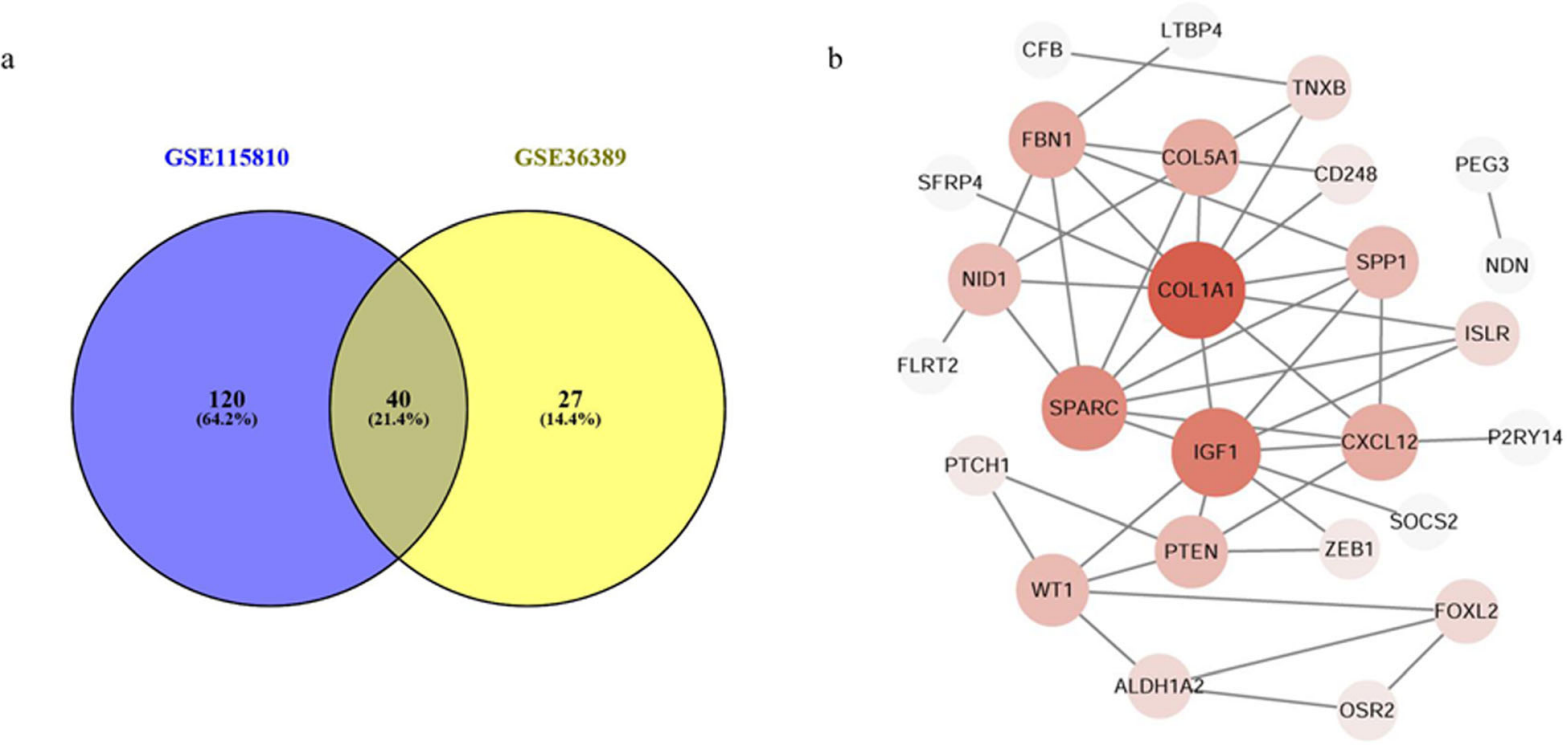
Identification of the DEGs. **a** Venn diagrams for the identification of DEGs in endometrial carcinoma (EC); 40 genes were overlapped in GSE115810 and GSE36389, and then were identified as DEGs. **b** Protein-protein interaction (PPI) network for the DEGs

Based on the GEO database and GEO2R online analysis tool, a total of 160 DE mRNAs were detected from GSE115810, of which 13 were upregulated mRNAs and 137 were downregulated; a total of 67 DE mRNAs were detected from GSE36389, of which 15 were upregulated and 52 were downregulated (Fig. 1). Subsequently, these two groups of DE mRNAs were intersected and there were 40 mRNAs which overlapped between GSE115810 and GSE36389. Finally, these 40 overlapped mRNAs, with 3 upregulated and 37 downregulated, were filtered out and identified as the DEGs of EC (Fig. 2a).

### PPI network construction and hub genes identification

A PPI network of the DEGs was constructed by STRING and visualized by Cytoscape to further explore the relationships among these genes. According to the node pair combing score ≥ 0.4, a total of 26 DEGs were filtered into the PPI network that interacted with other DEG(s) (Fig. 2b). Furthermore, according to the topological properties analysis of the PPI network, several nodes have higher connectivity degree, with degree ≥ 5 set as the criterion. These nodes were Collagen alpha-1(I) chain (COL1A1, degree = 11), insulin-like growth factor 1 (IGF1, degree = 9), secreted protein acidic and rich in cysteine (SPARC, degree = 8), collagen alpha-1(V) chain (COL5A1, degree = 6), fibrillin-1 (FBN1, degree = 6), CXC chemokine ligand 12 (CXCL12, degree = 6), phosphatase and tensin homolog (PTEN, degree = 5), secreted phosphoprotein 1 (SPP1, degree = 5), and Wilms tumor 1 (WT1, degree = 5). Besides, most of these nodes could interact with each other in the PPI network.

### GO term and KEGG pathway enrichment analysis of DEGs

**Fig. 3:**
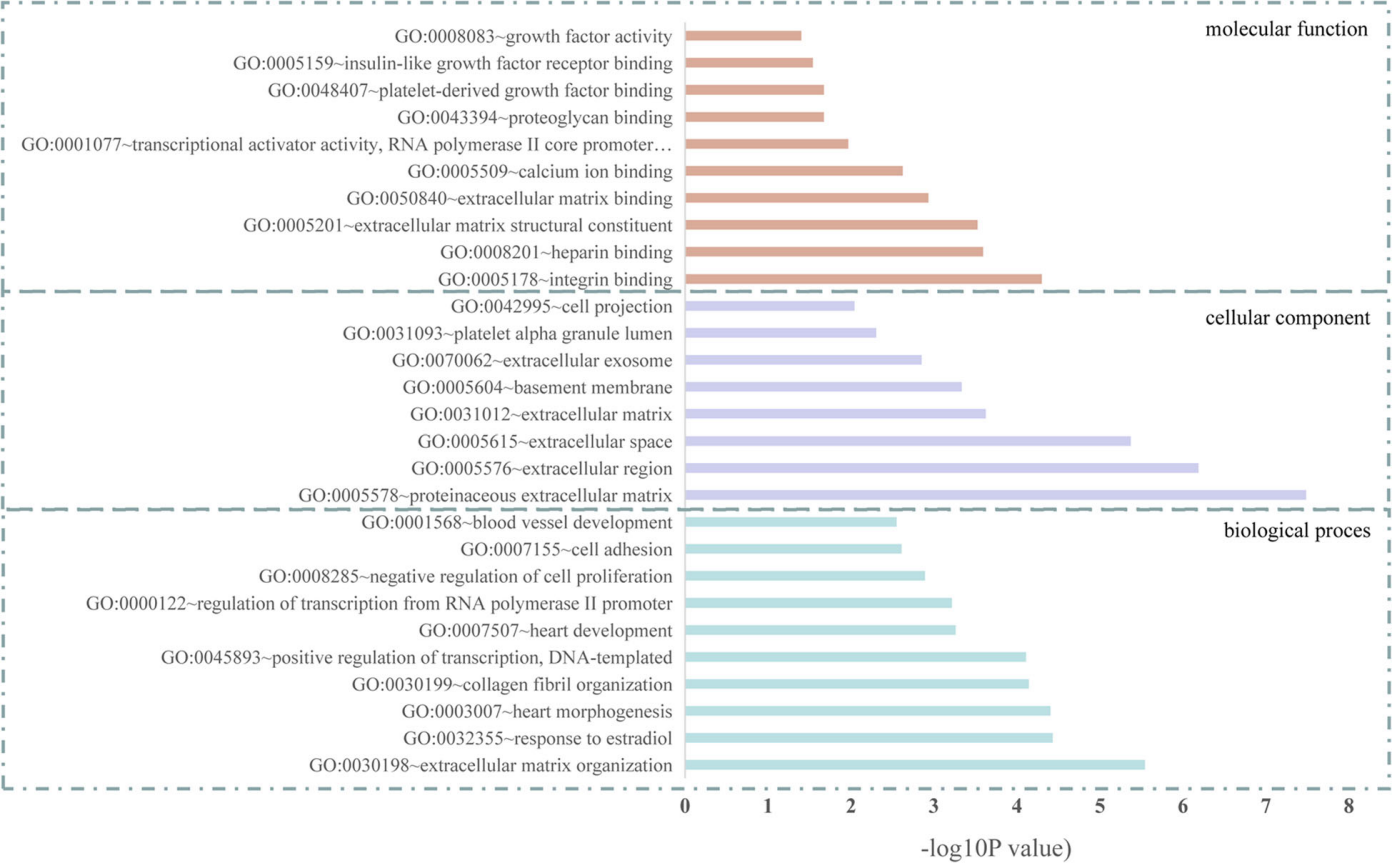
The gene ontology (GO) enrichment analysis of the DEGs in EC. GO were divided into three categories: biological process, cellular component, molecular function. Each enriched GO terms of DEGs were ranked by − log10 (*P* value)

**Fig. 4:**
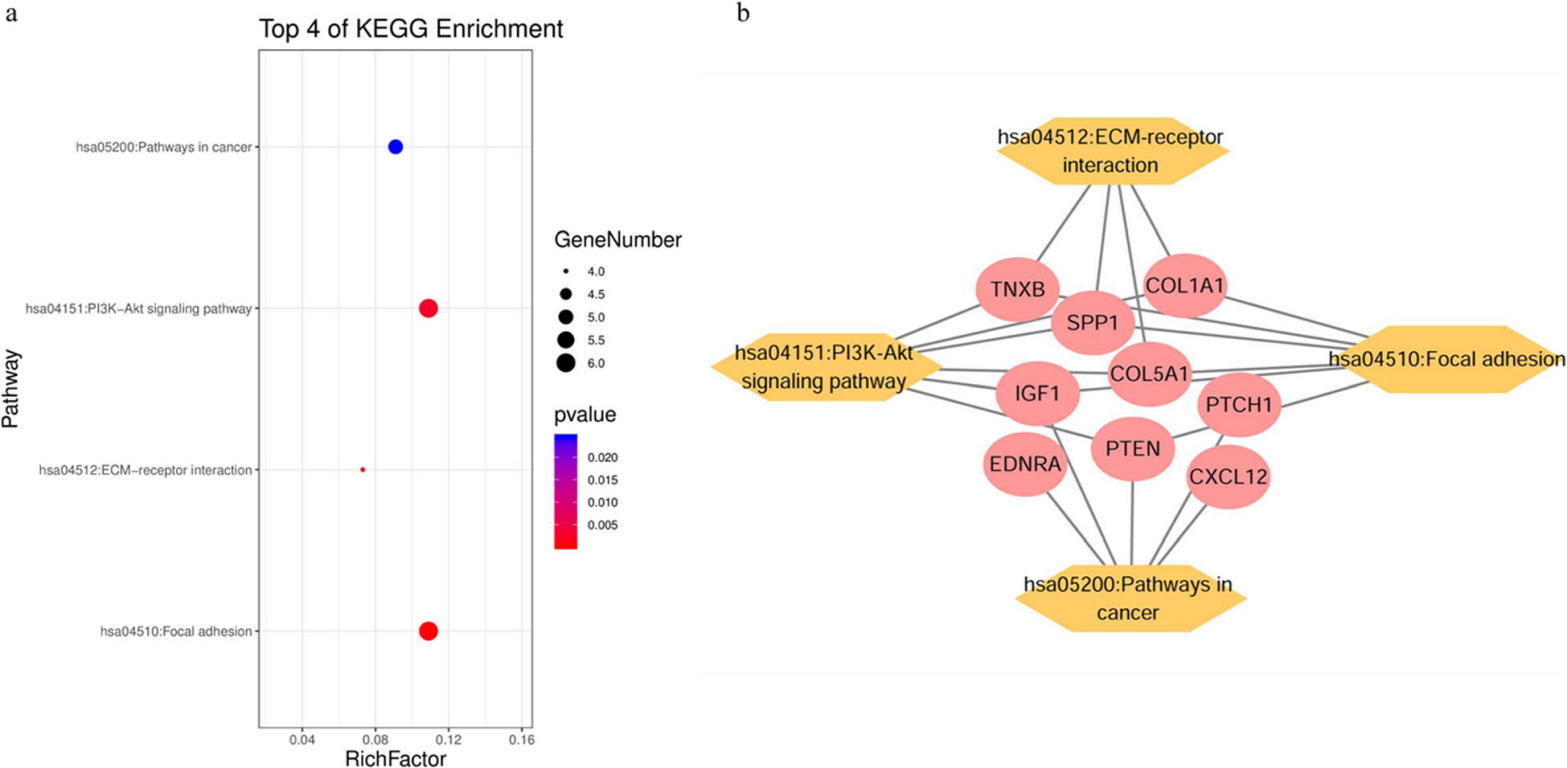
Kyoto Encyclopedia of Genes and Genomes (KEGG) pathway analysis of the DEGs. **a** KEGG pathway of the DEGs. **b** DEG-KEGG pathway network

GO and KEGG enrichment analysis were performed to gain further insight of the bioinformatics information of the 40 DEGs (Table 1). The each enriched GO term of DEGs was ranked by − log_10_ (*P* value) (Fig. 3). GO terms includes biological processes, cellular components, and molecular function. The significant enriched biological process terms were extracellular matrix (ECM) organization, response to estradiol, collagen fibril organization, negative regulation of cell proliferation, cell adhesion, blood vessel development, positive regulation of epidermal cell differentiation/apoptotic process, cell migration, etc.; the significantly enriched cellular component terms were proteinaceous ECM, extracellular region, ECM, basement membrane, extracellular exosome, platelet alpha granule lumen, and cell projection; and the significantly enriched molecular function terms were integrin/heparin binding, ECM structural constituent, ECM binding, calcium ion binding, insulin-like growth factor receptor binding, etc. KEGG pathways enrichment analysis of the DEGs suggests that focal adhesion, ECM-receptor interaction, PI3K-Akt signaling pathway, and pathways in cancer were found to be significantly enrichment pathways (Fig. 4a). Moreover, for the multiple genes involved in the identified KEGG pathways, the DEG-pathway network showed that some of the DEGs exhibited active participations, including COL1A1, IGF1, COL5A1, PTEN, CXCL12, and SPP1 (Fig. 4b). Former PPI analysis also identified these genes with higher node degree, then these six DEGs were considered as hub DEGs and utilized for further analysis.

**Table 1:** GO term enrichment analysis of DEGs

Term	*P* value	Genes
Biological process		
GO:0030198~extracellular matrix organization	2.87E−06	CRISPLD2, FBN1, NID1, COL1A1, SPARC, COL5A1, SPP1
GO:0032355~response to estradiol	3.72E−05	ALDH1A2, SOCS2, PTCH1, COL1A1, PTEN
GO:0003007~heart morphogenesis	3.95E−05	FLRT2, ALDH1A2, PTCH1, COL5A1
GO:0030199~collagen fibril organization	7.20E−05	TNXB, TNXA, COL1A1, COL5A1
GO:0045893~positive regulation of transcription, DNA-templated	7.81E−05	FOXL2, OSR2, GDF7, IGF1, PTCH1, COL1A1, TBL1X, WT1
GO:0007507~heart development	5.49E−04	EDNRA, FBN1, SPARC, PTEN, WT1
GO:0000122~negative regulation of transcription from RNA polymerase II promoter	6.09E−04	FOXL2, OSR2, PTCH1, FOSB, ZEB1, TBL1X, WT1, PEG3
GO:0045944~positive regulation of transcription from RNA polymerase II promoter	7.55E−04	FOXL2, OSR2, NDN, IGF1, FOSB, ZEB1, TBL1X, WT1, PEG3
GO:0008285~negative regulation of cell proliferation	1.29E−03	ALDH1A2, NDN, SFRP4, ZEB1, PTEN, WT1
GO:0007155~cell adhesion	2.46E−03	ISLR, TNXB, COL1A1, CXCL12, COL5A1, SPP1
GO:0001568~blood vessel development	2.83E−03	ALDH1A2, COL1A1, COL5A1
GO:0001658~branching involved in ureteric bud morphogenesis	3.45E−03	PTCH1, DCHS1, WT1
GO:0043434~response to peptide hormone	3.78E−03	COL1A1, SPARC, CXCL12
GO:0051591~response to cAMP	4.12E−03	COL1A1, FOSB, SPARC
GO:0009612~response to mechanical stimulus	6.70E−03	PTCH1, FOSB, CXCL12
GO:0030324~lung development	1.09E−02	ALDH1A2, CRISPLD2, SPARC
GO:0022617~extracellular matrix disassembly	1.09E−02	FBN1, NID1, SPP1
GO:0032964~collagen biosynthetic process	1.24E−02	COL1A1, COL5A1
GO:0045606~positive regulation of epidermal cell differentiation	1.45E−02	SFRP4, PTCH1
GO:0008284~positive regulation of cell proliferation	1.55E−02	ALDH1A2, OSR2, CD248, IGF1, PTEN
GO:0071711~basement membrane organization	1.66E−02	FLRT2, NID1
GO:0032836~glomerular basement membrane development	1.86E−02	NID1, WT1
GO:0002576~platelet degranulation	1.94E−02	ISLR, IGF1, SPARC
GO:0048853~forebrain morphogenesis	2.07E−02	GDF7, PTEN
GO:0048048~embryonic eye morphogenesis	2.07E−02	FOXL2, FBN1
GO:0060322~head development	2.07E−02	EDNRA, OSR2
GO:0006366~transcription from RNA polymerase II promoter	2.13E−02	FOXL2, OSR2, NDN, FOSB, WT1
GO:0043065~positive regulation of apoptotic process	2.42E−02	ALDH1A2, FOXL2, SFRP4, WT1
GO:0042493~response to drug	2.51E−02	PTCH1, COL1A1, FOSB, PTEN
GO:0007417~central nervous system development	2.58E−02	NDN, ZEB1, PTEN
GO:0014032~neural crest cell development	2.68E−02	EDNRA, ALDH1A2
GO:0001501~skeletal system development	3.30E−02	FBN1, IGF1, COL1A1
GO:0048738~cardiac muscle tissue development	3.49E−02	ALDH1A2, PTEN
GO:0008283~cell proliferation	4.02E−02	EDNRA, IGF1, ZEB1, PTEN
GO:0017015~regulation of transforming growth factor beta receptor signaling pathway	4.09E−02	LTBP4, ZEB1
GO:0007411~axon guidance	4.32E−02	FLRT2, GDF7, CXCL12
GO:0016477~cell migration	4.98E−02	CD248, PTEN, COL5A1
Cellular component		
GO:0005578~proteinaceous extracellular matrix	3.29E−08	FLRT2, TNXB, TNXA, CRISPLD2, CD248, LTBP4, FBN1, SPARC, COL5A1
GO:0005576~extracellular region	6.52E−07	CFB, GDF7, LTBP4, FBN1, IGF1, NID1, SPARC, PTEN, CXCL12, COL5A1, ISLR, CRISPLD2, SFRP4, COL1A1, SPP1
GO:0005615~extracellular space	4.26E−06	FLRT2, TNXB, GDF7, CFB, LTBP4, SFRP4, FBN1, IGF1, COL1A1, SPARC, CXCL12, RAMP1, SPP1
GO:0031012~extracellular matrix	2.38E−04	TNXB, LTBP4, FBN1, NID1, COL1A1, COL5A1
GO:0005604~basement membrane	4.64E−04	FBN1, NID1, SPARC, COL5A1
GO:0070062~extracellular exosome	1.41E−03	FLRT2, TNXB, TNXA, CFB, LTBP4, CD248, FBN1, NID1, CXCL12, SLC7A5, COL5A1, ISLR, CRISPLD2, SPP1
GO:0031093~platelet alpha granule lumen	4.99E−03	ISLR, IGF1, SPARC
GO:0042995~cell projection	9.11E−03	NDN, PTEN, SPP1
Molecular function		
GO:0005178~integrin binding	5.03E−05	TNXB, LTBP4, FBN1, IGF1, COL5A1
GO:0008201~heparin binding	2.57E−04	TNXB, CRISPLD2, FBN1, PTCH1, COL5A1
GO:0005201~extracellular matrix structural constituent	2.99E−04	TNXB, FBN1, COL1A1, COL5A1
GO:0050840~extracellular matrix binding	1.17E−03	CD248, SPARC, SPP1
GO:0005509~calcium ion binding	2.39E−03	SLC24A3, CD248, LTBP4, FBN1, NID1, SPARC, DCHS1
GO:0001077~transcriptional activator activity, RNA polymerase II core promoter proximal region sequence-specific binding	1.08E−02	FOXL2, NDN, FOSB, WT1
GO:0043394~proteoglycan binding	2.13E−02	NID1, COL5A1
GO:0048407~platelet-derived growth factor binding	2.13E−02	COL1A1, COL5A1
GO:0005159~insulin-like growth factor receptor binding	2.89E−02	SOCS2, IGF1
GO:0008083~growth factor activity	3.98E−02	GDF7, IGF1, CXCL12

### Genetic alterations of the hub genes in EC

**Fig. 5:**
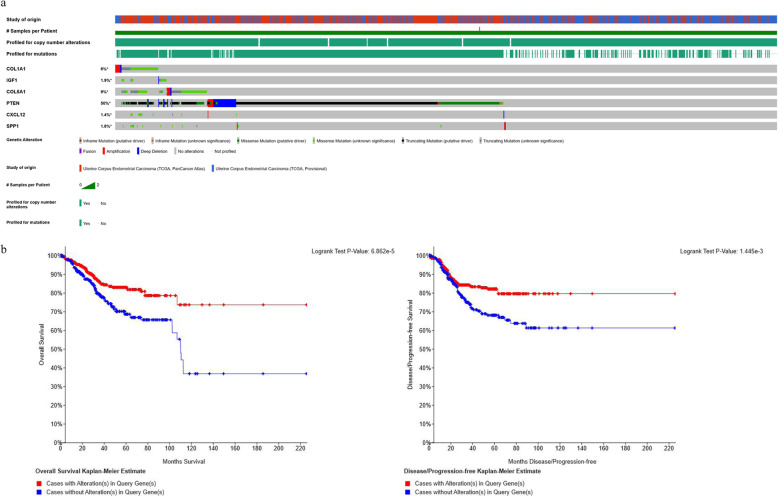
Genetic alterations and survival analysis of the total six hub genes in EC by cBio portal. **a** A visual summary of alteration across a set of EC samples (data taken from the TCGA Data Portal) based on a query of the six genes (COL1A1, IGF1, COL5A1, CXCL12, PTEN, and SPP1). Distinct genomic alterations are summarized and color-coded, presented by % changes in particular affected genes in individual tumor samples. Each row represents a gene and each column represents a tumor sample. **b** Overall survival and the disease-free survival rates of the EC patients with or without COL1A1, IGF1, COL5A1, CXCL12, PTEN, and SPP1 mutations. The red curves in the Kaplan-Meier plots include cases with gene alteration, and the blue curves include cases without gene alteration

cBio Portal online database was applied for a further investigation of these hub genes in EC oncogenesis. Genetic alterations of hub genes and their cancer clinical profiles in EC samples were observed from the TCGA database based on cBio Portal platform. As shown in Fig. 5a, tumor samples with sequencing and CNA data for each gene also implied that 562 cases (52%) of 1076 sequenced cases had alterations in at least one of the hub DEGs queried. For COL1A1, IGF1, COL5A1, CXCL12, PTEN, and SPP1, the genetic alteration rate were 6%, 1.9%, 9%, 1.4%, and 1.8%, respectively. When it comes to PTEN, it showed that half of the cases were altered (50%) and the alterations were classified as truncating mutation, missense mutation, deep deletion, frame mutation, and amplification.

The survival results indicated that these total six DEGs were also obtained from the cBio Portal platform. Overall survival and disease-free survival were compared between cancer cases that have alteration of these queried genes and without alteration of these genes in EC samples (Fig. 5b). For the overall survival, cases without alteration in the hub genes have worse survival than cases with alteration, and significant difference was found with a *P* value of 6.862E−05 < 0.05. For the disease-free survival, the outcome consists of the overall survival, with a *P* value of 1.445E−03 < 0.05.

### Correlations between COL1A1 and IGF1, COL5A1, PTEN, CXCL12, and SPP1

**Fig. 6:**
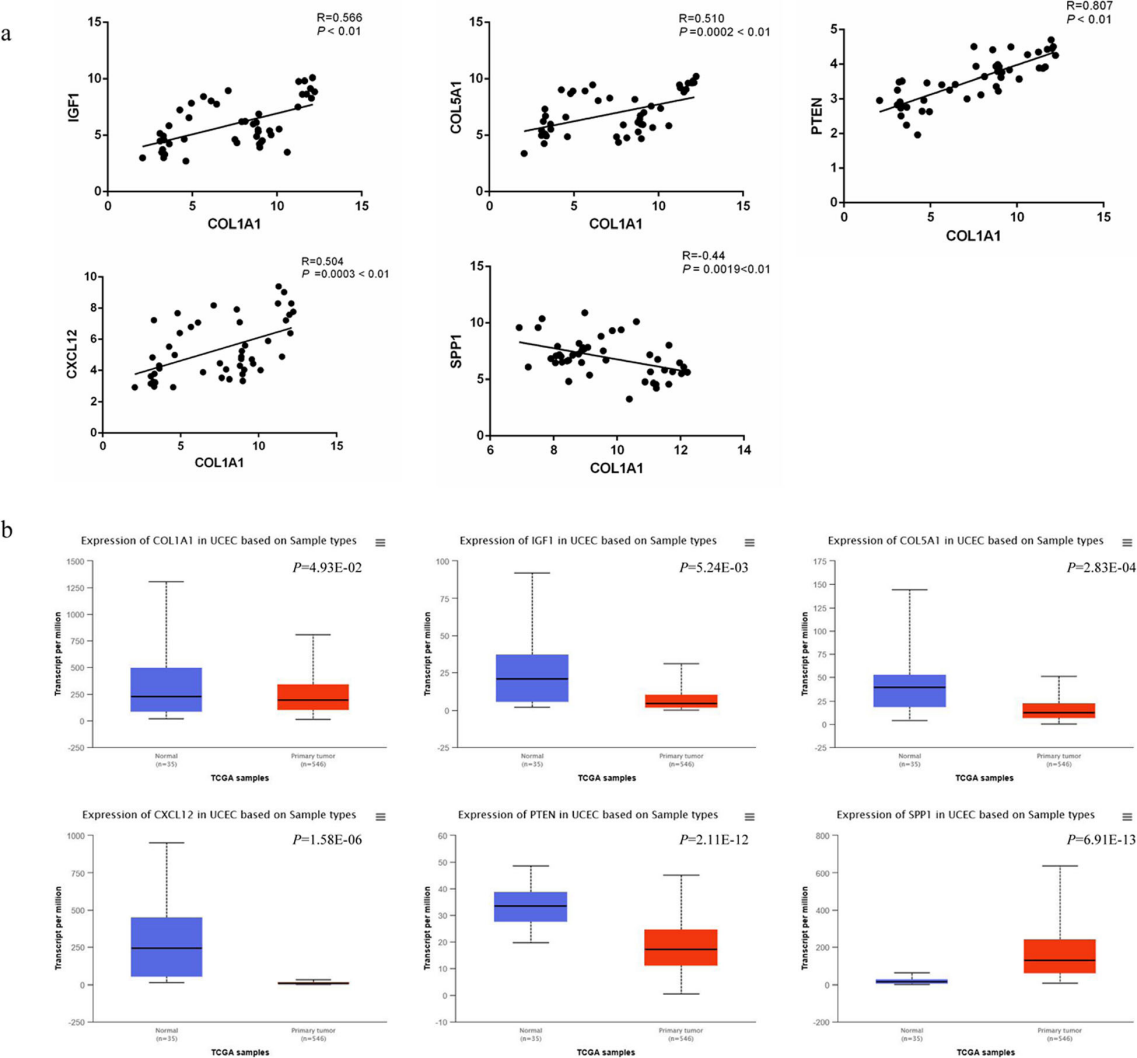
**a** Correlation analysis between COL1A1, IGF1, COL5A1, CXCL12, PTEN, and SPP1. **b** Expression profiles of COL1A1, IGF1, COL5A1, CXCL12, PTEN, and SPP1 in EC patients based on TCGA samples. The blue box represents normal samples; red box represents tumor samples

According to the raw data of the gene expression profiles in GSE115810 and GSE36389, the correlations between COL1A1 and IGF1, COL5A1, PTEN, CXCL12, and SPP1 were analyzed. As shown in Fig. 6a, the results indicated that there were significant correlations between COL1A1 and IGF1 (*R* = 0.566, *P* < 0.01), COL5A1 (*R* = 0.510, *P* < 0.01), PTEN (*R* = 0.807, *P* < 0.01), CXCL12 (*R* = 0.504, *P* < 0.01), and SPP1 (*R* = 0.44, *P* < 0.01), respectively.

### Expression validation of COL1A1, IGF1, COL5A1, CXCL12, PTEN, and SPP1 in EC

**Fig. 7:**
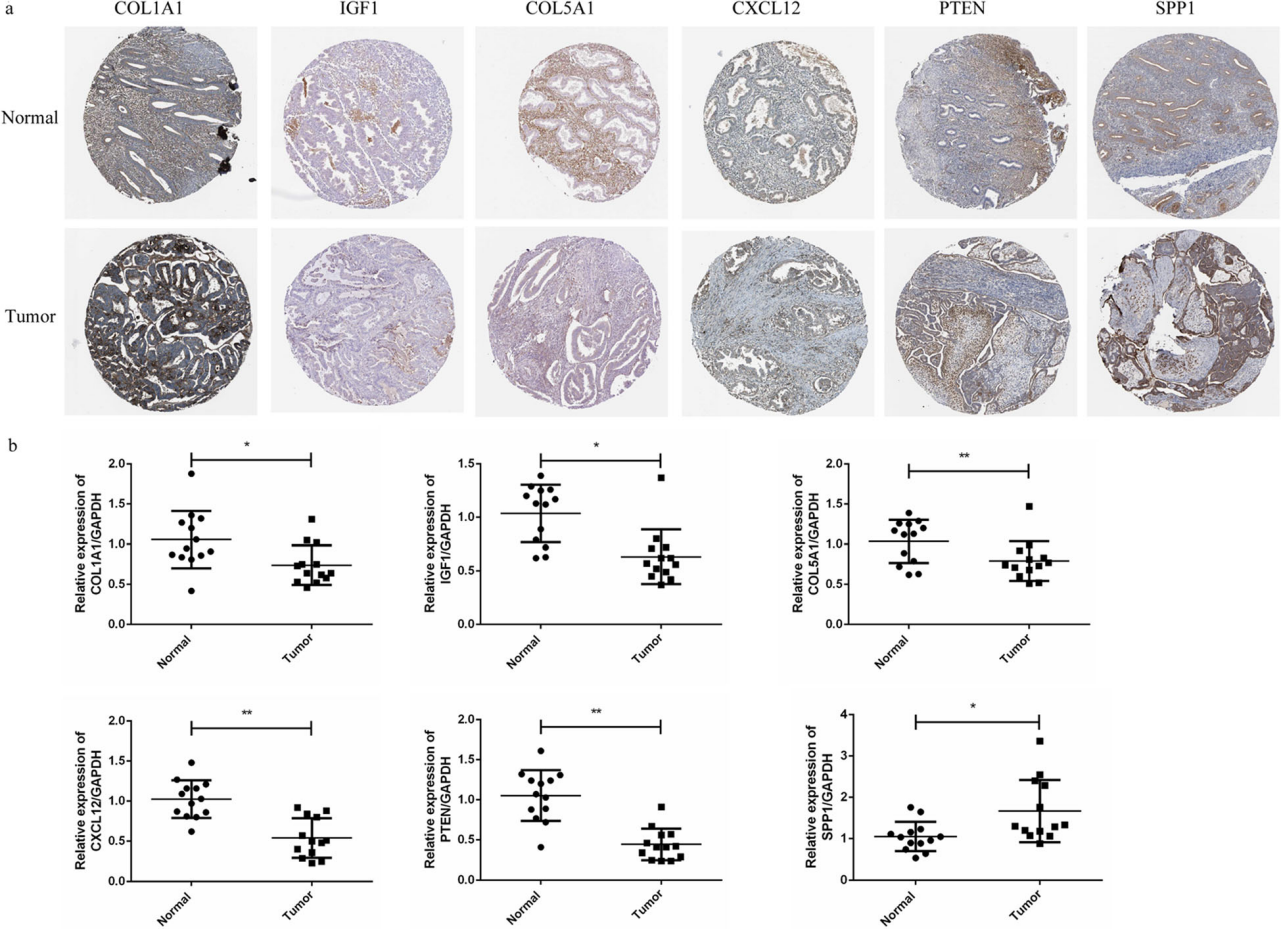
**a** Immunohistochemistry map of the hub genes protein expression in normal and EC tissues. **b** qRT-PCR validation of the expression of COL1A1, IGF1, COL5A1, CXCL12, PTEN, and SPP1 in 13 pairs of samples form EC patients and adjacent normal samples. *P < 0.05, **P < 0.01

Due to the former analysis found that hub DEGs were frequently mutated in EC patients and related with EC survival, we made further analysis on these genes. Based on TCGA platform and online tool UALCAN, the expression profiles of hub DEGs in EC were further validated. As shown in Fig. 6b, it indicated that the COL1A1, IGF1, COL5A1, CXCL12, PTEN, and SPP1 were significantly differently expressed in EC tumor compared with those in normal samples (*P* = 4.93E−02, *P* = 5.24E−03, *P* = 2.83E−04, *P* = 1.58E−06, *P* = 2.11E−12, and *P* = 6.91E−13, respectively). Furthermore, the immunohistochemistry map of the hub genes protein expression in normal and EC tissues was also shown in Fig. 7a, and different expressions of these hub genes in normal and EC tissues also could be observed. The mRNA expression of these hub DEGs were also verified using qRT-PCR in 13 pairs of samples form EC patients and adjacent normal samples. qRT-PCR results showed that the mRNA expression of COL1A1, IGF1, COL5A1, CXCL12, PTEN, and SPP1 were also significantly differently expressed in normal samples and tumor samples (Fig. 7b). The relative expression of COL1A1, IGF1, COL5A1, CXCL12, and PTEN in tumor samples was significantly lower compared to those in adjacent normal samples (*P <* 0.05), while SPP1 expression was significantly higher in tumor samples compared to the adjacent normal samples (*P <* 0.05).

### Prognosis values of the COL1A1, IGF1, COL5A1, CXCL12, PTEN, and SPP1 in EC

**Fig. 8:**
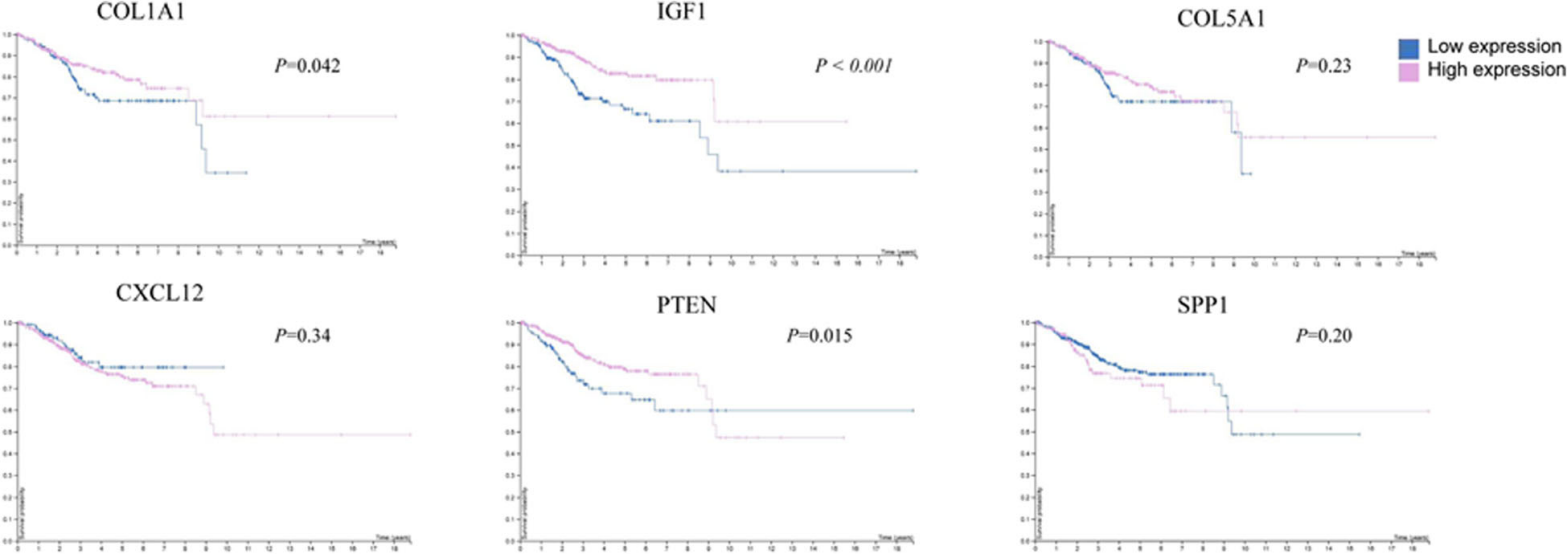
Survival analysis of COL1A1, IGF1, COL5A1, CXCL12, PTEN, and SPP1 in EC. *P* < 0.05 was considered as statistically significant. The blue line represents the low expression of DEG; red line represents the high expression of DEG

Prognosis value of the each hub gene was also analyzed with the survival analysis. As shown in Fig. 8, the Kaplan-Meier survival analysis showed that the survival outcome in high expression of COL1A1, IGF1, and PTEN patients was significantly better than low expression patients (*P* = 0.042, *P* < 0.001, *P* = 0.015, respectively).

## Discussion

EC, as common gynecological malignant tumors posing serious threat to the women health worldwide, its pathophysiological process remains elusive and complicated. In present study, bioinformatics analysis was performed based on the gene expression microarray dataset of GSE115810 and GSE36389 from the GEO database. A total of 40 genes were screened out as the EC related DEGs. Functional enrichment analysis showed that these DGEs were significantly enriched in biological processes including extracellular matrix (ECM) organization, response to estradiol, collagen fibril organization, negative regulation of cell proliferation, cell adhesion, blood vessel development, positive regulation of epidermal cell differentiation/apoptotic process, cell migration, etc.; KEGG pathways include focal adhesion, ECM-receptor interaction, PI3K-Akt signaling pathway, and pathways in cancer. The functional enrichment evidence strongly shored up the supposition that these DEGs can affect the proliferation, differentiation, migration, and apoptosis of EC cells through the aforementioned biological processes and pathways and thus regulate the onset and progression of EC. More importantly, COL1A1, IGF1, COL5A1, CXCL12, PTEN, and SPP1 were identified as hub DEGs with potential values in EC.

In this study, the expression of COL1A1 and COL5A1 were downregulated in EC samples compared with normal samples in microarray datasets and further expression validation. Survival analysis also found that the survival outcome of EC patients in high expression of COL1A1 was significantly better than that in low COL1A1 expression patients (*P* = 0.042). And in 1076 sequenced EC cases, the genetic alteration rate of COL1A1 was 6% and COL5A1 was 9%; the major genetic alterations were amplification, deep deletion, and missense mutation. COL1A1 and COL5A1 all belong to the collagen family; this family is the major component of the tumor microenvironment, it interacts with substances in the ECM (such as matrix metalloproteinases), integrins, tyrosine kinase receptors, and some signaling pathways to influence tumor cell behavior and activity [11, 12]. Evidencing studies of collagen in diverse cancers illustrate the dual roles of collagen. Study found that COL1A1 gene expression was markedly decreased in hepatocellular carcinoma tumor tissues (log_2_ ratio − 1.1) with a poor overall survival rate (*P* = 0.013) [13]. Tumorigenesis of EC is a complex process, with multiple oncogenes and anti-oncogenes critically involved, forming a tight connected network, affecting multiple signaling pathways and metabolism or biological processes in the organs, and thus inducing the progression of tumor. PPI network in the present study showed that these genes could mostly interact with each other; correlation analysis also revealed that the expression of COL1A1 and the rest of hub genes IGF1, COL5A1, CXCL12, PTEN, and SPP1 was significantly correlated in EC. This indicated that these genes have tight correlations with others, and these correlations might amplify their roles in EC.

Periodic changes in IGF1 expression play a key role in regulating the transition of premenopausal endometrium through procreation, secretion, and menstrual cycle. In this study, IGF1 was screened as a hub DEG of EC, expression validation in TCGA database and clinical samples proved that IGF1 was downregulated in EC samples, and survival analysis also indicated that the EC patients with high IGF1 expression have better survival outcome than those low expression group patients (*P* < 0.001). IGF1 is thought to be a potential mediator for the effects of estradiol on uterine growth. Epidemiological, clinical, and experimental data also shore up the evidence that IGF1 is an important players in general gynecological cancers, and particularly in endometrial tumors [14–16]. The downregulation of IGF-1 was also found in endometrial cancer specimens compared to the adjacent normal specimens in Soufla’s study, which seems to comprise the main features of endometrial carcinogenesis [17]. CXCL12 expression is also associated with the survival of cancer patients, but the role is dual. High CXCL12 expression was associated with reduced overall survival in patients with esophagogastric (*P* = 0.002), pancreatic (*P* = 0.0005), and lung cancer (*P* = 0.01), whereas in breast cancer patients, high CXCL12 expression conferred an overall survival advantage (*P* < 0.001) [18]. Felix et al. suggest that the positive CXCL12 expression was associated with longer overall survival (log-rank *P* = 0.006) and longer recurrence-free survival (log-rank *p* = 0.01) in estrogen receptor (ER) negative EC patients, but not in ER positive EC patients [19]. Another study found that the expression of CXCR4 was predominant (*P* = 0.035) whereas CXCL12 was low-expressed (*P* = 0.002) in EC [20].

It is proved that PTEN produces tumor suppressor effect mainly through blocking the PI3K/Akt signaling pathway based on its lipid phosphatase activity, thus inhibiting the activity of Akt and inducing the apoptosis of tumor cells [21]. This is consistent with our KEGG enrichment results that PTEN and other hub DEGs were mainly involved in the PI3K/Akt signaling pathway. Hyperactivation of PI3K/Akt signaling is the risk factors for carcinogenesis of cells, and it is regulated by a variety of factors, whose negative feedbacks are mainly regulated including PTEN [22]. As a tumor suppressor gene, the lost or mutation of PTEN was revealed and regarded as diagnostic biomarker in multiple types of cancers, like prostate cancer [23], breast cancer [24], gastric cancer [25], cervical carcinoma [26], and EC [27, 28]. In our study, microarray analysis and expression validation found that PTEN was downregulated in EC samples; survival analysis also indicated that the EC patients with high PTEN expression have better survival outcome than those low expression group patients (*P* < 0.015). SPP1, also called osteopontin, is involved in various cellular processes like ECM binding, osteoblast differentiation, cell adhesion, immune regulation, and ECM receptor interaction acting through interaction with multiple cell surface receptors including CD44 [29, 30]. Except for the critical role in wound healing and chronic inflammatory diseases, the expression of SPP1 also has strong relationship with numerous tumors. Abnormal expression of SPP1 was found in numerous cancers and also has potential correlation with cancer prognosis [31, 32]. Our study showed that in 1076 sequenced EC cases, the genetic alteration rate of SPP1 was 1.8%, and the major alteration was missense mutation. Tu et al.’s study also observed that mutations in SPP1 primarily occurred in cutaneous melanoma and endometrial cancer, with the ratio of alteration which ranged from 1.03 to 9.23% [33].

Genetic alterations results in this study implied that half of the EC cases (52%) had alterations in at least one of the hub DEGs queried, with the genetic alteration rate varied from 1.4 to 50% for hub genes, especially for PTEN alteration. It showed that half of the cases were altered (50%) and the alterations were classified as truncating mutation, missense mutation, deep deletion, frame mutation, and amplification. Gene mutation is responsible for the abnormal functions of the code protein and might further cause abnormal phenotype, including tumor occurrence. Mutations of anti-oncogene were often observed in variety of tumors, and this may correlated to the acceleration of tumorigenesis [34]. PTEN is the most commonly and frequently mutated gene in EC, and the loss of PTEN is considered to be an early event of endometrial tumorigenesis. In this study, further overall and disease-free survival analysis observed statistically different outcomes with these DEG alterations, and individual survival analysis of hub genes in EC patients also demonstrated that low expression of COL1A1, IGF1, and PTEN were related with poor survival outcomes in EC patients, which indicated that these DEGs might critically correlate with the EC prognosis.

## Conclusion

In conclusion, the present study identified six genes (COL1A1, IGF1, COL5A1, CXCL12, PTEN, and SPP1) with crucial role in tumorigenesis and progression in EC; our results suggested these genes could add a new dimension to our understanding of the EC and might be served as potential biomarkers that will be assisting cancer biologists and clinical oncologists in developing novel therapeutic strategies for EC patients. However, there are some limitations in this study. Further larger clinical sample size and in-depth experiment studies in vivo and in vitro are needed to clarify the clear mechanism and warrant the prognostic value of these DEGs in EC.

## Data Availability

The datasets supporting the conclusion of this article are included within the article.

## Abbreviations

EC: Endometrial carcinoma
DEGs: Differentially expressed genes
GEO: Gene Expression Omnibus
GO: Gene ontology
KEGG: Kyoto Encyclopedia of Genes and Genomes
PPI: Protein-protein interaction network
TCGA: The Cancer Genome Atlas
COL1A1: Collagen alpha-1(I) chain
IGF1: Insulin-like growth factor 1
SPARC: Secreted protein acidic and rich in cysteine
COL5A1: Collagen alpha-1(V) chain
FBN1: Fibrillin-1
CXCL12: CXC chemokine ligand 12
PTEN: Phosphatase and tensin homolog
SPP1: Secreted phosphoprotein 1
WT1: Wilms tumor 1
ECM: Extracellular matrix
IRS1: Insulin receptor substrate 1
MAPK: Mitogen-activated protein kinases
CXCR4: CXC motif chemokine receptor 4
SHIP2: SH2-containing inositol 5-phosphatase
PIP3: Plasma membrane intrinsic protein 3
